# Case Report: High-dose pregabalin ingestion in an adolescent: clinical course and serial plasma concentrations

**DOI:** 10.3389/ftox.2026.1822851

**Published:** 2026-06-25

**Authors:** Raffaele Simeoli, Enrico Armiento, Sara Cairoli, Mara Pisani, Paola Silvestri, Marco Marano

**Affiliations:** 1 Laboratory of Metabolic Diseases and Drug Biology, Bambino Gesù Children’s Hospital, IRCCS, Rome, Italy; 2 Residency School of Pediatrics, University of Rome Tor Vergata, Rome, Italy; 3 Emergency Unit, Bambino Gesù Children’s Hospital, IRCCS, Rome, Italy; 4 Pediatric Poison Control Center, Bambino Gesù Children’s Hospital, IRCCS, Rome, Italy; 5 Pediatric Intensive Care Unit, Bambino Gesù Children’s Hospital, IRCCS, Rome, Italy

**Keywords:** clinical toxicology, conservative management, extracorporeal treatment indications, pediatric intoxication, pregabalin overdose

## Abstract

**Background:**

Pregabalin (PGB) is widely used for neuropathic pain, generalized anxiety disorder, and seizure management; however, recent epidemiological data highlight a progressive increase in its misuse and involvement in intoxications, particularly among adolescents and individuals with psychiatric comorbidities. Although dose–toxicity relationships have been explored in adult populations, substantial interindividual variability persists, and data in pediatric or adolescent patients remain limited. Establishing clearer associations between ingested dose, serum concentrations, and clinical severity is essential to guide management strategies and identify cases requiring advanced interventions such as extracorporeal removal.

**Case Presentation:**

We describe the case of a 16-year-old girl who intentionally ingested 2,700 mg (54 mg/kg, based on the patient’s body weight of 51 kg) of immediate-release pregabalin. She presented at the Emergency Care Unit 10 h after ingestion with marked somnolence, psychomotor slowing, and bilateral horizontal nystagmus, while vital signs, renal and hepatic function tests remained normal. Electrocardiography revealed only mild PR interval prolongation. Serial plasma concentrations of Pregabalin demonstrated a decline from 16.5 mg/L at 12 h to 8.4 mg/L at 18 h and 0.69 mg/L at 34 h, consistent with expected pharmacokinetics. No gastrointestinal decontamination was performed due to delayed presentation, and no extracorporeal treatments were deemed necessary. Patient was subjected to a clinical conservative management, and progressively recovered without neurological sequelae.

**Conclusion:**

Despite ingestion of a dose commonly associated with moderate to severe toxicity, the patient exhibited a mild and self-limited clinical course, emphasizing the variability of pregabalin toxicity in adolescents. Clinical assessment remains the primary guide, while serum concentrations provide supportive information when available.

## Background

Pregabalin is widely prescribed for neuropathic pain, generalized anxiety disorder, and partial-onset seizures. Recent epidemiological data highlight increasing global misuse, intoxications, and pregabalin-associated fatalities, particularly in patients with psychiatric comorbidities or concomitant opioid use (Evoy KE et al., 2021). However, the dose–toxicity correlation remains highly variable among individuals, and the clinical significance of plasma pregabalin concentrations is still debated. This Case report contributes to understanding the relationship between dose, plasma concentration, and clinical toxicity in adolescent patients.

## Case presentation

A 16-year-old girl intentionally ingested 2,700 mg (54 mg/kg, based on the patient’s body weight of 51 kg) of immediate-release pregabalin (Lyrica®). Ten hours post-ingestion, she presented to the Emergency Department with marked somnolence, psychomotor slowing and occasional bilateral horizontal nystagmus. A more complete neurological assessment indicated slurred speech and a Glasgow Coma Scale score of 15. The patient reported active suicidal ideation (C-SSRS 6/6), leading to the activation of the continuous observation protocol. No vomiting, seizures, gait disturbances, or severe behavioural changes were reported. Vital sings always remained stable and she had no need for ventilatory and hemodynamic support.

Baseline laboratory tests were unremarkable, showing normal creatinine (comprised between 0.65–0.75 mg/dL) and hepatic transaminases (ALT 8 U/L - AST 13 U/L). Venous blood gas analysis showed a mild, transient lactate elevation (2.9 mmol/L) that rapidly normalized, and an initial mild reduction in glucose which also resolved (83 mg/dL).

Electrocardiography revealed only mild PR interval prolongation (200 m, compared with 167 m on a prior ECG performed 9 months earlier) which resolved within 48 h.

The patient described in this Case was previously admitted to our Emergency Department in March 2025 following the intentional ingestion of approximately 30 tablets of paracetamol without informing her parents. Patient was subsequently admitted to the Child and Adolescent Neuropsychiatry Unit of our Institution, where previous diagnoses (January 2025) of bulimia nervosa, dysthymia, and attention-deficit/hyperactivity disorder (ADHD), predominantly inattentive presentation, were confirmed. In this occasion, continuation of pharmacological treatment with aripiprazole (7.5 mg twice daily) and Lithium sulphate (166 mg once daily) was recommended.

Based on the patient’s current treatment, lithium serum levels were measured resulting within the therapeutic range (0.70 mEq/L).

Our patient was not under treatment with PGB, but she has found tablets among father’s medications. Therefore, she did not receive any prescription for it. Hence, tolerance phenomena can be excluded.

### Investigations

PGB concentrations in plasma samples were measured using a validated CE/IVD LC-MS/MS kit (MassTox® TDM Series A Antiepileptic Drugs in Serum/Plasma) provided by Chromsystems (Chromsystems Instruments and Chemicals GmbH, Germany). Liquid chromatography and mass spectrometry analyses were performed on a UPLC ACQUITY I Class coupled to a Xevo® TQ-S micro-triple quadrupole mass spectrometry system (Waters™ Corporation, United States). The calibration curve for PGB was linear over the range of 0.15–12.9 mg/L. Samples resulting in a concentration above the highest calibration (ULOQ) point were diluted using pooled blank plasma and re-analyzed.

**FIGURE 1:**
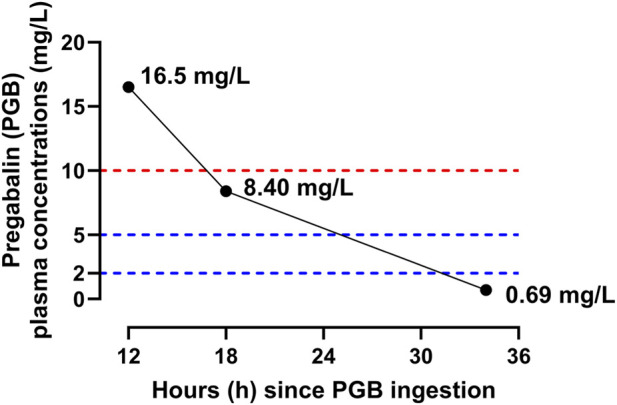
Pregabalin (PGB) plasma concentrations measured at 12, 18, and 34 hours post-ingestion. Horizontal dashed blue lines indicate PGB therapeutic range whereas dashed red line represents the laboratory alert threshold.

After 12 h (h) from ingestion, a plasma measurement of PGB was performed showing a level of 16.5 mg/L. Plasma samples were further collected at 18 and 34 h following PBG ingestion and pregabalin concentrations were 8.4 and 0.69 mg/L, respectively (Figure 1). The established therapeutic range for PGB is 2–5 mg/L, whereas the laboratory alert value is 10 mg/L (Hiemke C et al., 2018).

In our hands, the observed PGB values showed a decline consistent with the known pharmacokinetics of pregabalin, which displays rapid absorption, with peak levels reached within 1.5 h (up to 3 hours with food), high oral bioavailability, negligible metabolism, and almost exclusive renal elimination (Tjandrawinata RR. et al., 2015). Its short half-life (4.6–6.8 h) generally supports a self-limited course in healthy individuals and provides the rationale for reserving extracorporeal removal for severe toxicity or renal impairment (Bouchard J et al., 2022).

### Differential diagnosis

Alternative diagnoses were also considered. A toxicology first level screening was performed by LC-MS/MS on urine samples collected at the Emergency Department. The comprehensive toxicological screening included the following analytes: fentanyl; ethyl glucuronide (EtG); barbiturates; benzodiazepines; methadone; opiates; buprenorphine; 6-monoacetylmorphine (6-MAM); cocaine and its metabolite benzoylecgonine; amphetamines; methamphetamine; cannabinoids (Δ9-tetrahydrocannabinol, THC); tricyclic antidepressants (TCAs); oxycodone; tramadol; alpha-pyrrolidinopentiophenone (α-PVP, also referred to as “flakka”); AB-PINACA; synthetic cannabinoids (JWH-018 and UR-144). A negative result was observed for all the analyzed compounds, indeed a confirmatory quantitative method was not performed. Moreover, post-ictal state or seizure-related etiology (unsupported by clinical features), metabolic disturbances (laboratory results were normal), intoxication with other gabapentinoids were also excluded.

The overall presentation was consistent with isolated pregabalin overdose.

### Treatment

Given the delayed presentation (>10 h) and the immediate-release PGB formulation, activated charcoal was deemed unlikely to provide benefits. Moreover, since pregabalin is not subjected to enterohepatic circulation, repeated doses of activated charcoal were not administered.

The patient was managed with symptomatic and supportive care, which included continuous cardiac monitoring for the initial 48 h, neurological reassessments (every 15 min), intravenous glucose–saline fluids (Normosol 60 mL/h for approximately 27.5 h), and symptomatic treatment only.

No antidotes or extracorporeal elimination techniques were required. These interventions are reserved for cases involving coma, hemodynamic instability, impaired renal function, or significant sedative co-ingestion.

Dose-toxicity patterns suggest a continuum of risk: mild symptoms typically occur with <20 mg/kg ingested, moderate toxicity between 20 and 50 mg/kg, and severe outcomes (including CNS depression and occasional conduction abnormalities) above 50–60 mg/kg (Rietjens SJ et al., 2022).

Pediatric studies propose an observation threshold near to 20 mg/kg for predicting clinically significant effects. However, interindividual variability and co-ingestants limit the reliability of dose alone. Likewise, serum concentration–toxicity relationships are debated: coma has been reported at 60–70 mg/L, symptoms at ∼30 mg/L, and concentrations near to 20 mg/L may occur without overt toxicity (Elliott SP et al., 2017).

### Outcome and follow-up

Over the next 24–36 h, the patient improved progressively: nystagmus resolved, alertness normalized, ECG remained stable and the initial mild PR prolongation (200 m) fully resolved within 48 h, plasma pregabalin levels declined predictably. The patient was discharged following 48 h of medical observation without neurological sequelae. In light of the intentional nature of the ingestion and the pediatric age group, she was immediately transferred to the Child and Adolescent Neuropsychiatry Unit of our Hospital for specialized management of her pre-existing comorbidities, including Bulimia Nervosa, Dysthymia, and ADHD. She was discharged after 11 days and enrolled in a treatment program at the community mental health services for ongoing clinical and psychiatric monitoring. She also underwent follow-up at our hospital’s pharmacotoxicology outpatient clinic 10 days after discharge. Follow-up visits included an ECG, blood tests for liver, pancreatic, thyroid, and renal function, a lipid profile, a complete blood count, a fasting blood glucose level, a coagulation profile, and serum lithium levels measurement.

## Discussion

This case adds to the current literature by highlighting the complexity of pregabalin toxicity assessment, particularly in pediatric patients. In our patient, a high ingested dose (54 mg/kg) and plasma concentrations exceeding the laboratory alert threshold were associated with only mild and self-limited symptoms, supporting the concept of marked interindividual variability and the limited predictive value of dose and serum levels alone. Moreover, the 12-h plasma level (16.5 mg/L) suggests that higher concentrations were likely present earlier, yet no severe toxicity developed. She presented with only mild neurological depression. The availability of serial plasma measurements further contributes to understanding the dose–concentration–effect relationship in a real-world setting, which remains insufficiently characterized in younger populations. Importantly, the favourable clinical course in the absence of specific interventions reinforces a conservative management approach in clinically stable patients with preserved renal function, suggesting that extracorporeal removal should be reserved for patients presenting with coma, refractory hemodynamic instability, impaired renal function, or significant sedative co-ingestion (Bouchard J, et al., 2022).

However, due to marked interindividual variability in pregabalin response, clinical assessment should integrate dose (when known), PGB levels measurement, symptomatology, potential polydrug exposure and comorbidities. Therefore, a conservative management appears appropriate in patients with normal renal function, preserved renal and hepatic function (confirmed by normal creatinine and transaminases), stable vital signs and no co-ingestion of sedatives. Moreover, since our patient was not under treatment with PGB, tolerance phenomena can be excluded.

The delayed hospitalization (10 hours post-ingestion) could represent the main limitation of this Case presentation. In fact, since pregabalin is rapidly removed in healthy subjects, the delay likely reduced the opportunity to observe maximal pharmacodynamic effects in presence of higher peak concentrations. However, it is worth to say that following pregabalin ingestion, the patient had attempted to vomit unsuccessfully and then went to bed without informing her parents. In the morning, awakened by her usual alarm clock, she informed her parents, who then took her to the Emergency Department. Therefore, we were not able to assess the possible concentration-related toxicity of PGB before patients’ arrival at our attention.

In conclusion, this Case demonstrates that high-dose pregabalin ingestion in adolescents may result in mild, self-limited toxicity. Therefore, clinical assessment remains the primary guide for patients’management, whereas serum concentrations, when available, should be regarded as supportive information. Finally, our Report provides a more comprehensive perspective for clinical decision-making in pregabalin overdose, although further data are required to define a clinically meaningful plasma threshold and to guide management decisions on the use of extracorporeal techniques in pediatric patients.

## Data Availability

The original contributions presented in the study are included in the article/supplementary material, further inquiries can be directed to the corresponding author.

## References

[B1] Bouchard J. Yates C. Calello D. P. Gosselin S. Roberts D. M. Lavergne V. (2022). Extracorporeal treatment for Gabapentin and pregabalin poisoning: systematic review and recommendations from the EXTRIP workgroup. Am. J. Kidney Dis. 79 (1), 88–104. 10.1053/j.ajkd.2021.06.027 34799138

[B2] Elliott S. P. Burke T. Smith C. (2017). Determining the toxicological significance of pregabalin in fatalities. J. Forensic Sci. 62 (1), 169–173. 10.1111/1556-4029.13263 27864947

[B3] Evoy K. E. Sadrameli S. Contreras J. Covvey J. R. Peckham A. M. Morrison M. D. (2021). Abuse and misuse of pregabalin and Gabapentin: a systematic review update. Drugs 81 (1), 125–156. 10.1007/s40265-020-01432-7 33215352

[B4] Hiemke C. Bergemann N. Clement H. W. Conca A. Deckert J. Domschke K. (2018). Consensus guidelines for therapeutic drug monitoring in neuropsychopharmacology: update 2017. Pharmacopsychiatry 51 (1-02), 9–62. 10.1055/s-0043-116492 28910830

[B5] Rietjens S. J. Sikma M. A. Hunault C. C. de Lange D. W. Hondebrink L. (2022). Pregabalin poisoning: evaluation of dose–toxicity relationship. Br. J. Clin. Pharmacol. 88 (3), 1288–1297. 10.1111/bcp.15073 34505299 PMC9293434

[B6] Tjandrawinata R. R. Setiawati E. Putri R. S. Gunawan V. A. Ong F. Susanto L. W. (2015). Pharmacokinetic equivalence study of two formulations of the anticonvulsant pregabalin. Clin. Pharmacol. 7, 69–75. 10.2147/CPAA.S82143 25945069 PMC4408967

